# Exploring the lived experience of receiving mental health crisis care at emergency departments, crisis phone lines and crisis care alternatives

**DOI:** 10.1111/hex.14045

**Published:** 2024-04-08

**Authors:** Helena Roennfeldt, Nicole Hill, Louise Byrne, Bridget Hamilton

**Affiliations:** ^1^ Centre for Mental Health Nursing University of Melbourne Melbourne Victoria Australia; ^2^ Department of Social Work University of Melbourne Melbourne Victoria Australia; ^3^ School of Management RMIT University Melbourne Victoria Australia; ^4^ Program for Recovery and Community Health, Department of Psychiatry Yale School of Medicine New Haven Connecticut USA

**Keywords:** crisis, crisis alternatives, crisis care, crisis phone lines, emergency department, mental health

## Abstract

**Background:**

Mental health crisis care includes emergency departments (EDs), crisis phone lines and crisis alternatives. Currently, there is an overreliance on EDs to provide mental health crisis care, with evidence that responses are often inadequate to meet the needs of people experiencing mental health crises. However, the complexities of how individuals experience crisis care across the varying contexts of EDs, crisis phone lines and crisis alternatives remain underresearched.

**Method:**

This study used a hermeneutical phenomenological approach to understand the lived experience of accessing care during a mental health crisis. Thirty‐one Australian adults who had accessed crisis services at ED, phone lines and/or crisis alternatives participated in in‐depth interviews.

**Results:**

The findings are organised across the temporal narratives of participants' experiences from (1) point of contact, (2) positive and negative care experiences and (3) enduring impacts. Several themes were generated during these phases. The findings demonstrate the interrelated nature of care experiences and enduring impacts. With some exceptions, care received within EDs was harmful, resulting in lasting adverse effects. Responses from crisis phone lines were mixed, with participants appreciating the accessibility of after‐hour phone support but finding standardised risk assessments unhelpful. Responses from crisis alternatives to ED were promising, aligning with the need for validation and human connection, but were not always accessible or easy to mobilise during a crisis. Notably, across all settings, positive effects were firmly attributed to the quality of the relationship with specific crisis providers.

**Conclusion:**

The findings bring into sharp focus the lived experience of people accessing crisis care and contribute to the shortage of literature on subjective experiences. Providers may better meet the needs of those experiencing mental health crises by understanding the enduring impact of these interactions and the role of human connection beyond a focus on risk assessment, thereby providing opportunities for a joint understanding of risk and meaning‐making. Furthermore, understanding the subjective experience of crisis care can guide reforms to ED and develop crisis alternatives to better meet the needs of people in crisis.

**Patient or Consumer Contribution:**

The first author and the third author are in designated lived experience (Consumer) roles. The first author conducted the interviews and was explicit regarding their lived experience when engaging with participants. Service users were involved as advisors to the study and provided input into the design.

## 1 INTRODUCTION

Mental health crisis care can be seen as a critical social and public health issue, with current crisis care described as inconsistent and inadequate.^1^ The extent of the failure of present systems to meet the needs of people is evident in high mental health presentations to emergency departments (EDs) in Australia and internationally.^2^, ^3^, ^4^ In 2020–2021, there were approximately 300,000 mental health presentations to Australian public EDs, comprising 3% of all ED presentations.^5^ The most frequent presentations were related to stress, psychoactive drug use and schizophrenia.^6^ The ED is also an important point of contact for people who are suicidal or who have self‐injured, with claims that reported rates are grossly underrepresented.^7^ Significantly, compared to physical health presentations, people with mental health concerns are much more likely to arrive by ambulance or police.^6^

Although EDs are often seen as the only available option, crisis phone lines also have a decisive role in providing help to people in crisis, particularly suicide prevention.^8^ More recently, to address the shortfall of EDs, there has been increasing implementation of crisis alternatives to EDs, including nonresidential options, such as the Safe Haven Café, first implemented in the United Kingdom,^9^ and residential facilities, such as the Living Room model developed in the United States.^10^ These approaches recognise that the ED is frequently poorly equipped to meet the needs of people in mental health crises.^11^ In Australia, a recent rollout of Safe Spaces based on the Safe Haven model is yet to be evaluated.^12^ Alternatives generally use nonclinical approaches that do not involve psychiatric diagnosis or treatments^10^ and employ peer workers who provide support that draws on their first‐hand experience of emotional distress using relationship‐based approaches, offering hope and connection.^13^ Entirely peer‐operated alternatives have also been developed as nonclinical and noncoercive approaches, committed to peer values of choice and mutual support, such as Alternatives to Suicide (Alt2Su) support groups.^14^

A mental health crisis is not easy to define but is broadly conceived of as the presence of emotional distress beyond the coping mechanisms of the individual.^15^ However, crisis services involving emergency services and the ED operate within a regulatory framework linked to mental health legislation and provision for involuntary treatment.^16^ Within this crisis care response, a mental health crisis is often more narrowly understood in reference to the laws of involuntary treatment involving risk to self and others.^17^ There is mounting evidence of the negative impact of involuntary treatment on future help‐seeking.^18^ More broadly, ineffective responses to mental health crises in EDs are well documented, including long wait times and poor outcomes.^19^, ^20^, ^21^ The responses received in the ED may even exacerbate the mental health crisis, contributing to maintaining a cycle of crisis and repeated presentations to the ED, sometimes referred to as ‘frequent flyers’ or people repeatedly accessing the ED in need of acute support.^22^

To date, the limited qualitative literature on how crisis care is experienced has been predominantly situated within EDs,^23^, ^24^ providing significant knowledge on experiences of crisis specific to this context, particularly challenges accessing ED. There is limited evidence for the effectiveness of crisis phone lines, and little is known about what factors influence their value and how this is measured.^25^ Similarly, findings on the effectiveness of alternative crisis models are limited, and it is unknown what factors have contributed to the success and challenges of these approaches.^12^ Still, findings indicate that alternative models to ED provide important advantages over traditional service delivery in relation to satisfaction and measures of personal recovery.^9^, ^10^, ^26^, ^27^

Relatively few studies have examined the lived experience of mental health crisis care more deeply and taken a hermeneutical phenomenological approach to understanding these experiences. Such an approach offers considerable value by prioritising subjective meaning and interpretation^28^ by elevating the perspectives and voices of people with first‐hand experience of crisis and what support is helpful in resolving their crisis. The present study, therefore, aims to build on existing literature on crisis care by taking a phenomenological approach to understanding the lived experience of crisis care within the contexts of ED, crisis phone lines or an ED alternative for people experiencing a mental health crisis.

## 2 METHOD

### 2.1 Study design

The philosophical positioning for this study drew from the interpretive Heideggerian phenomenological tradition.^29^, ^30^ This approach holds that the meaning of experiences is socially constructed and interpreted through an embodied and subjective lens situated within a sociocultural and geographic context. Using in‐depth interviews, we invited participants to narrate their experience of crisis and the crisis care that they received. Crisis care was defined as the initial contact with ED, phone lines and alternatives.

Ethics was approved by the University of Melbourne Human Research Ethics Committee (2021‐21024‐21395‐4), and the participants were provided with both written and verbal information before agreeing to participate. Names of the participants given in the findings are pseudonyms (assigned by the researcher or chosen by participants) or participants' first names, depending on preference.

### 2.2 Participants

Participants were eligible to participate if they were aged over 18 years, spoke English, had accessed ED, crisis phone lines or crisis alternatives during a mental health crisis and were open to speaking about their experiences. Purposive recruitment employed maximum variation sampling to capture a breadth of crisis and crisis care experiences^31^ to ensure varied representation in age, gender, period since accessing crisis services and types of support accessed. Given that the study explored experiences over three different contexts, the sample size needed to allow for this diversity.

The study was promoted on social media and via email through mental health networks. Prospective participants contacted the first author, where they received further information and were screened for eligibility. Eligibility was confirmed with all individuals before they were invited into the study and gave their informed consent. Before their involvement in the study, ethical considerations were also discussed with participants, including potential distress in discussing experiences of crisis care and the identification of personal supports and strategies for self‐management. Participants were also provided with contact numbers if they wished to follow up on any concerns raised during the interview. All participants were reimbursed with an e‐gift card of $100.

### 2.3 Data collection

In‐depth interviews were conducted via Zoom videoconferencing (*n* = 27), in person (*n* = 2) or by phone (*n* = 2), as preferred by participants. The interviews were conducted from July to October 2021. During this time, Australia was considerably impacted by social distancing measures due to coronavirus disease 2019 outbreaks, which made face‐to‐face interviews problematic. Interviews began by introducing each other and then discussing the research goals and expectations. Demographic and background information was collected via email or in the initial screening call.

Interviews elicited a participant's narrative, which allowed the participant to remember a past event and recount it in light of what is meaningful for them. The opening question: Can you describe the point where you received mental health crisis care? was intentionally broad to allow participants to guide the direction of the interviews and what was significant to their individual experiences. Additional guiding questions and prompts were flexibly used to encourage deep and multifaceted reflections.

The first author conducted all interviews and was transparent in their identification as someone with personal experience accessing crisis services. Research conducted by people with lived experience has been found to facilitate more in‐depth data than traditional methods,^32^ and disclosure was used to generate greater equality and trust within the interview. In alignment with the phenomenological approach, the researcher was committed to listening deeply to identify what people care about and provide insight into participants' underlying beliefs and interpretations.^33^

### 2.4 Data analysis

Interviews were audio‐recorded, transcribed verbatim and coded using Atlas‐ti software. In line with centring lived experience, participants were provided with a narrative summary of their interview to check and confirm the accuracy of their account of their experience. Reflexive thematic analysis was used to analyse the data,^34^ taking an inductive approach to theme generation, driven bottom‐up by the expressed lived experience of participants. The first author immersed themself in the transcripts to ensure familiarity with the complete data set before coding. The approach to coding involved ongoing discussion of potential themes developed from the data with the second and last author and repeated refinement of codes.

## 3 RESULTS

The final sample comprised 31 participants aged between 18 and 75 years (mean = 42, SD = 13). Most participants identified as female (20), with a smaller number identifying as male (7), nonbinary (2) and transgender (2). Two interested individuals who approached regarding participation were ineligible; one decided not to participate; and two were unresponsive when later contacted to arrange the interview. Although smaller sample sizes are standard in phenomenology (approximately *n* = 10), a larger sample was needed to address the study's aims, with the richness of the data taking precedence over the actual size of the sample.^31^

For participants, the crisis experience was described as self‐injury, suicidality, psychosis and overwhelming emotions resulting from grief, past trauma or other life situations. The demographic questions revealed that the vast majority of participants identified having accessed ED (*n* = 30), and the number of presentations ranged from participants who had accessed ED once to participants who had been to ED over 100 times; 25 participants had used crisis phone lines; and 15 participants had accessed crisis alternatives. The types of crisis alternatives that participants had accessed covered Safe Spaces that employed peer workers alongside clinical staff, a peer‐operated respite service, Alt2Su peer support group, a private wellness retreat, psychologists and a private hospital that could adopt more alternative and holistic approaches.

During the interview, participants narrated accounts of their crisis care experience, with most choosing to share their experiences in ED (*n* = 30), with a smaller number discussing crisis lines (*n* = 8) and alternative crisis services (*n* = 8). The themes are organised across the temporal narrative of participants' experiences: (1) point of contact, (2) positive and negative care experiences and (3) enduring impacts. Several themes were generated within these overarching sections. Notably, the themes were not universally experienced across experiences of ED, crisis phone lines and crisis alternatives, with dismissive and delegitimising responses applicable primarily to crisis care within ED and phone lines. Where relevant, the themes are presented first from the experience of ED, then crisis phone lines and lastly, alternatives, with a description of each alternative discussed.

### 3.1 Point of contact

Accessing crisis care was marked by the inaccessibility of care, invisibility and inability to express emotional pain, a need for safety and involvement of emergency services. Initially, when accessing ED, participants talked about there being no other option and nowhere to turn.

#### 3.1.1 Nowhere to turn

Participants spoke of feeling destitute, that there was nowhere to turn to get help and nothing available to ‘catch’ the crisis earlier, ‘I know that there's nothing to catch those extreme states, so I try and push it away as much as possible’ (Morgan).

You know, I do feel desperate and really need help. I don't know what else to do. So, I rock up to the ED. And I basically get told, oh, well, if you want to kill yourself, go ahead and do it. We're not going to stop you. (Fiona)

Before both suicide attempts, I tried talking to people around me to say, hey, I'm really not coping, but not getting the kind of response that I needed. Or, I suppose, maybe not being taken seriously for the risk that it posed. (Tyler)

Concerning crisis phone lines, participants were grateful to be able to reach someone by phone after hours when the usual support systems were inaccessible.

Sometimes, it's too hard to actually talk to someone face to face about that kind of thing, and because quite often, it happens at night‐time anyways, when there's no support worker or psychologist or anything like that available, so crisis lines come into play then. (Crystal)

Alternatives were not always available and, at times, had restrictions and conditions governing accessibility that did not match the needs of participants. The following example is from safe spaces.

They're trying to do the Safe Havens here. But in our area, they're only going to do a youth one. And I'm like, why are we only doing youth—you're eliminating all the older people who need something in our area? So, there is nothing really where I live. (Helen)

#### 3.1.2 Invisibility of emotional pain

For all participants, the challenges in conveying their crisis were marred by a need to make visible or express the invisibility of their emotional pain in a way that others could ‘see’ to respond. Many participants felt that their despair was increased by an expectation held by crisis services that they could articulate their experience in accessing crisis services. Yet, for many participants, their crisis experience could not be put into words or expressed in a way others could understand.

When I'm in a crisis, I find it very hard to talk and say what I'm actually feeling and very hard to reach out because I can't articulate what it is. I am too far down that black hole to just say anything. (Di)

I know that a lot of what I would have said to people at the time might not have made sense. To the average person and to medical people, they would have sounded quite mad. But I think, no, I know, it did make sense. (Blair)

Participants battled to express their experiences in words. They felt that they were seen as not needing attention because they lacked a shared understanding of their experience within a clinical interpretation, ‘I need to self‐harm. Yeah, I need to attempt suicide. I need to injure myself first before I warrant attention. Well before they will believe me’ (Amy).

#### 3.1.3 Need for safety

Regardless of where participants accessed crisis care, the point of contact was linked to the need for safety and the recognition of the life‐threatening potential of their situation, ‘I did call triple zero [Australian emergency services phone contact] because I felt, you know, this is an emergency, this is this is life and death’ (Gregory)

In the face of desperation and needing safety, some participants felt like they needed physical containment within the ED to be safe. Yet, participants recognised that this safety came at a cost.

In hindsight, it was containment 100% because I was at risk of harming myself, and I had really detailed plans. Like it was a justified response … . I think it's really interesting because, for me, I'm in two minds about it. Ironically, I view it as a positive thing. But then also it's a detrimental thing. (Cathy)

Safety through physical containment was recognised as a short‐term or Band‐Aid response. Participants were put in a position where they needed to advocate for their continued support.

The emergency department stopped me from putting my suicide plan into action for four nights. And then they were gonna say, ‘Well, it's nice to see you, but bye’, and I would have gone out and put my plan into action. (Clare)

#### 3.1.4 Involvement of police and ambulance

For several participants, receiving crisis care was initiated by others who perceived that they were at risk. For these participants, entry to crisis care was involuntary and involved an initial response and transport by police and ambulance services to the ED. Participants reported variable responses from police ranging from hostile to kind and caring.

The police would approach me, and they wouldn't ask questions or talk to me. They would just jump on me and beat me and handcuff me like I was a criminal. Like I was a violent criminal. And through those experiences, I am very, very frightened. … but there were two police officers [on one occasion] that were very good. Yeah, they just spoke to me and treated me like a person. Like a vulnerable, scared person. (Phoenix)

The majority of responses from ambulance services were reportedly positive for participants. However, there were also higher expectations of ambulance officers being caring in their responses, which made occasions when there were negative responses more jarring for participants.

I kind of expect the police, or I can justify it more in my head that the police are going to be assholes. I don't expect it, or I didn't expect it from paramedics. Yeah. I didn't expect it from ambulance. (Cathy)

The initial contact for each participant was characterised by the person feeling insufficient in themselves and finding that their existing relational supports and helping services around them were not enough. The participants' relationship with achieving safety was complex. For participants who wanted physical containment, this was perceived to stop them from ending their lives and only a short‐term response.

### 3.2 Positive and negative experiences of care

Participants described both positive and negative experiences of treatment and care. Overwhelmingly, participants described crisis responses in ED as unfavourable. However, negative experiences were also sometimes found when accessing crisis phone lines and rarely when accessing crisis alternatives.

#### 3.2.1 Expectations of care

Participant expectations shaped the experience of care. Some participants described the clash or mismatch of what they needed and what was offered. Participants recognised that EDs did not know what to do for people in emotional distress.

The experience of the emergency department was they had no idea what to do. They had no procedures, no protocols, nothing except putting someone in a bed for a short period of time because they did not understand the seriousness of it. If you're bleeding, or you've had a broken bone sticking out, or if you've got, you know, a diagnosable disease, they know exactly what to do. People in EDs are the most wonderful people, but you come in saying, I'm going to kill myself; they don't know what to do. (Clare)

I'll ring up, and I'll, you know, wait in line, and I'll do what I need to do. But I don't end up getting the help, and that's really problematic. And then I get frustrated with not receiving the help and being told you're not suicidal enough. (Amy)

#### 3.2.2 Dismissive and delegitimising responses

Dismissive and delegitimising responses related primarily to mental health crisis responses in EDs.

Too busy to care. Just, yeah, they let me stay the night and sent me home …so I stayed the night in the hospital. They said, ‘You've got a history of depression, blah, blah, blah. You've just had a baby. Okay, so it's probably postnatal depression. Just follow up with your GP’. (Leanne)

Participants' narratives likewise provide accounts of dehumanisation and othering within the ED.

A big thing that stands out to me was that it was dehumanising … I feel like I was an object of disgust. Like I was stitched up without anesthetic, and the doctor said, you know, I'm not gonna waste anesthetic on you … I felt like I wasn't a human being; I was a strange kind of disgusting thing to be dealt with. Blair

Generally, the treatment received for mental health was seen as different, and people described receiving less care than physical health presentations to ED.

It's jarringly different how we get treated if I have a physical ailment versus if I have a mental health ailment. Jarringly different how I get treated and it's the same hospital. When I present to ED with physical health, I'm not a waste of bed space. I'm not attention‐seeking, and I'm not wasting their time and their resources. (Amy)

At times, crisis phone lines were also identified as dismissive, ‘I have given up on Lifeline … they are the biggest letdown. Usually, the person on the other end is uninformed or misinformed, not compassionate and condescending’ (Rae).

When participants called crisis phone lines as the first point of contact, there was agreement that stock screening questions that were designed to assess risk were challenging and a deterrent to engaging with the crisis service, “It was, ‘Are you going to kill yourself? Yes or no. If you're going to kill yourself, I will call an ambulance’” (Helen).

These responses were viewed as focused on the responder's decisions and actions instead of taking time to listen and understand. Responses were often considered risk‐focused, quickly escalating the situation by calling emergency services. Although experiences of crisis alternatives were generally positive, there were instances where participants described alternatives that did not match the severity of their experience. The following account describes a pilot safe space.

The big focus was on safety planning. Safety planning was the big thing, and just giving you sort of, like, I think you got 45 minutes to sit there, calm down, get your safety plan, and you had to sort of leave within the hour sort of thing. (Fiona)

This predominance of safety planning without adequate consideration of individual experience and attention placed on relationships felt dismissive.

#### 3.2.3 Inappropriate responses to emotional distress

For some participants, the inappropriateness of the response in ED was reflected in being placed in seclusion for expressing their suicidality.

And then I felt this real cold, sharp, hostile, foreign environment in the ED … They put me in a room by myself and put a security guard on the room. It was very, very hostile in the way they treated me as if I was a risk, where all I had said is I need help, because I'm scared because I feel like I want to die. (Gregory)

Other participants felt that they needed to amplify their experience to receive care and compete to access resources.

I'm just here for a mental health thing. And I figured I'd have to up the ante if I wanted to get treated seriously. So, I had to come out and say, and I think my family jumped in and said that this man's just had a serious suicide attempt, and yeah, I felt a bit felt a bit unworthy and felt I had to talk my situation up and that felt a bit dirty. Yeah, you know, something I tried to hide for so long … and now almost to make it bigger to get some help. (Dale)

Overwhelmingly, the highly clinical environment of the ED was perceived as not designed for emotional pain and psychological distress that urgently needed care but did not need medical intervention. As a result, participants felt isolated in their crisis response experience. The experiences of these participants portrayed ED solely as a place for physical health needs, marginalising participants' experience of mental health crises, leaving a discord between the need for safety and care, with the reality of feeling dismissed and unworthy of care.

#### 3.2.4 Experiencing care through human connection

Across all crisis settings, the experience of care was felt through human connection. Human connection gave a sense of refuge characterised by compassion, acceptance and kindness. While many crisis responses in ED were negative, some positive experiences of care were noted, ‘They [clinicians in ED] did a little bit of self‐disclosure around how difficult they found work and life, and they were just very human’ (Morgan).

Conversely, the lack of human connection in ED often exacerbated the experience of crisis.

It's like, you know, being in the ocean, in the pitch black and getting tighter and tighter and tighter. And there's nothing to grab hold of. There's nobody there to help you. And you are just going under. Yeah, and her comment [clinician in ED] almost felt like she was, you know, smiling and just pushing my head under the water kind of thing. (Leanne)

Participants identified more helpful experiences and responses from crisis phone lines characterised by human connection.

I guess the one time when it was helpful was when I called Lifeline. And the person seemed to be really understanding and talked to me more about strategies, I guess. But she just, she really listened and acknowledged that, yes, I must be going through a hard time … Especially in needing to call the crisis line. And, yeah, that was really helpful. (Crystal)

Overall, alternatives were appreciated for being nonpathologising. Alt2Su was a peer support group for people who had personal experiences of suicidality and had the capacity to listen to people in distress and not respond by labelling or calling emergency services.

I've recently started going to an Alternative to Suicide group. And they've been wonderful. The ability to sit back and listen to you, the ability to hear each other, to take turns, to care without rushing to clinicalise you and rushing to assess you and risk assess you. (Amy)

Importantly, what constituted an ‘alternative crisis service’ was subjective, with another participant praising Alt2Su but recognising that for them, it was not a crisis service, ‘I go to the Alt2Su group. I've only been a couple of times. I only just sort of found out about it, and that's really good, but it's not a crisis service’ (Rae).

Similarly, peer‐run services provided more significant opportunities for human connection and equality than traditional clinical services, ‘The approach was different from a peer‐run organisation … just by the language he was using. I felt like he was speaking to me as a person’ (Alina).

The following participant describes how care and safety through human connection was established through emotional release and psychological containment.

I was listened to, accepted, and loved. And I was able to share what I have been carrying for so long. I had a time when I had so much sadness and tears, and I asked if I could just go into a room and cry, and I'd get a pillow and blanket, and I would just howl and howl. There was so much inside that needed release. (Emme)

Equally, individual therapists were valued for their creative and embodied practices, knowing that emotional experiences cannot be articulated or rationalised.

I like the embodied part of it, to actually get you to feel physically what that's like, so you're not just trapped in your head or thinking about big overwhelming feelings. It's all integrated together. Yeah, and not having to try and rationalise what you were doing. Because a lot of the time, these things don't make sense rationally. (Tyler)

Regardless of the context, positive interactions were characterised as ‘human‐to‐human’ responses. The psychological safety found in crisis alternatives was striking in contrast with the fragmented safety associated with being compulsorily restricted and physically contained.

### 3.3 Enduring impact of crisis care

Irrespective of the setting, participants described the enduring psychological impacts of crisis care often having lasting impacts on their sense of self, safety and future help‐seeking. Positive impacts from experiences within EDs included moments of connection and being heard that remained with people long after the experience, ‘The psychiatrist came along and sat there and cried with me. And that was, yeah, that was really lovely. I mean, I obviously haven't forgotten that moment’ (Matt).

However, more often, the negative impacts of accessing ED were identified by participants as losing their trust in themselves and others, with participants censoring their experience and being selective about who they shared with.

And so, the lasting impact is like selecting who I'm open with—and vetting them to make sure that there's enough trust there. And enough, like, felt sense of connection … So, I carry that five years later, don't tell doctors that you hear voices or that you are suicidal. So, even today, I am conscious of that. (Darcy)

Due to lingering negative impacts and the absence of alternatives, several participants stressed the importance of avoiding standard crisis responses and being alone and without help during their crisis experience.

I wouldn't go to the emergency department. And I have tried again in recent crisis times, the online crisis chat, it is painful and it's tough. Where I live, there isn't anything that I would use. And I guess I would just do what I've been doing, which is being alone with it. Morgan

In contrast, crisis alternatives were effective for some participants in providing a sustained sense of safety and reducing the need for crisis support.

It feels like a bit of a safety net that there's somewhere that I could say to people, I've been thinking about dying this week. And people, instead of being like, you should go to the hospital, they just say, ‘Oh, what's that been like, you know, what's going on?’ Like, ‘Why do you think that might have happened?’ It's more just like a normal discussion. (Amie)

Enduring impacts mirrored the quality of the experience within the crisis setting. Most participants reported enduring adverse effects of EDs that reflected the care as dismissive, delegitimising and dehumanising. In contrast to experiences at ED, most participants described positive experiences of alternatives as welcoming and inclusive spaces that obviated further need for crisis care in EDs of crisis phone lines.

## 4 DISCUSSION

This phenomenological study allowed participants to narrate their experience of crisis care in terms of what was most important to them. The findings followed care experiences during a mental health crisis from the initial point of contact to the experiences of care and lastly to the sustained impact of experiences. At the point of contact, the lack of perceived options and absence of support to ‘catch’ the crisis experience earlier reflect the well‐documented reliance on EDs.^35^ Desperation and attempts to reach out also reiterate the potential to circumvent the crisis before breaking point, reflected in the importance of time and timeliness in responding to a crisis.^36^

Findings support the accessibility of crisis phone lines as a first contact for people experiencing mental health crises and suicidality.^37^ Generally, accessing crisis support relied on people reaching out and being able to articulate their experience, and for many people, the crisis experiences intrinsically included difficulty in verbalising their needs.^38^ In the same way, as reflected by Fricker, participants felt that there was a restricted capacity for a shared understanding and more significant potential for hermeneutic injustice without shared ways to interpret their experience.^39^ Overall, findings support the need for crisis care as a place of being welcomed,^40^ and although experiences of crisis alternatives were positive,^11^ alternatives were not always available and sometimes challenging to mobilise during the crisis.

Concerning the crisis care received, the study adds weight to existing research showing a predominance of adverse mental health crisis care in EDs,^19^, ^20^, ^21^ with participants feeling dismissed and objectified based on their experience, which they felt was not considered as worthy of care as a physical health crisis. Through narrative accounts of responses in ED, participants portrayed mental health crises as the creation of the ‘other’. Through this process of dehumanisation, seemingly ‘normal’ people are assigned traits that make them figures of disgust or fear.^41^ People may also be depicted as less human because of the pervasive taboo surrounding suicide^42^ and fear of death.^43^ Using othering where people are set apart from the rest of society may reaffirm people's identity as separate and alleviate existential fear.^44^ This process of dehumanisation of people diagnosed with mental illness has been previously documented^45^ and associated with poorer treatment.^46^ Hence, people presenting with mental health crises felt unwelcome in ED, intensified and reinforced by adverse and involuntary treatment.

Experiences of crisis phone lines were mixed, as previously documented,^8^, ^25^ with the experience of standardised risk assessments, lack of personalised care and the likelihood of referral to emergency services most unhelpful. In contrast to experiences at ED, and aligned with early research on alternatives, most participants described positive experiences of alternatives as welcoming and inclusive spaces of emotional safety.^26^, ^27^ Emotional safety for participants in this study was characterised as an experience of human‐to‐human connection that was experienced as emotional holding that equated to a more healing response. Across all settings, more positive experiences were characterised by the quality of the caring relationships. This finding builds on previous literature on subjective experiences of accessing mental health care in the ED that revealed the mitigating role of relationships within the perception of care.^19^ This study extends this finding to include the relationship, including the quality of listening and connection, as a critical factor in the perception of care regardless of whether people accessed ED, crisis phone line or an alternative. This reflects the impact of relationships more broadly for people accessing mental health services and that it is the ‘emotional, not physical environment’ that is core to people's experiences.^47^

In contrast with a felt sense of safety provided relationally through emotional containment, physical measures of containment and involuntary treatment were expressed as measures of control used to provide physical but not psychological safety.^48^ Emotional containment refers to another person being available to help with complicated feelings and having the capacity to witness feelings too difficult for the person to manage on their own.^49^ Physical containment was sometimes perceived as justified as a means of providing safety. However, in line with existing research,^50^ coercive practices were more often seen to exacerbate the crisis experience and contribute to long‐lasting impacts, including the experience of dehumanisation and reluctance to seek help.

The highly emotional and vulnerable experience of crisis care often resulted in enduring impacts. Ultimately, some participants' negative impacts, unmet needs and retraumatisation prevented future crisis support engagement. For people accessing crisis care, the lack of alternatives and hampered capacity for relational responses to meet their need for human connection and safety was a fundamental source of negative experiences that impacted future help‐seeking. As a result, people relied more on their coping strategies and striving to manage alone, reflecting familiar barriers of fear, shame and stigma in seeking help.^51^

Possible limitations of the study include self‐nomination and the likelihood that participants were driven to participate by the desire to share more negative stories of crisis care. However, comparable adverse outcomes of ED for people experiencing mental health crises are supported by previous studies. Noting the size and diversity of the population accessing crisis services, it is acknowledged that this work was not designed to recruit a representative sample. As with all phenomenological research, we do not assert that these findings will generalise to other people and settings. Therefore, opportunities for others to take up these findings and conclusions depend on the extent to which they resonate with the reader. The study's strength is the authentic accounts of the complex phenomena of crisis care by trusting participants to share their perspectives of what was important in receiving crisis care. These findings contribute to a rich understanding of the experience of crisis care. Notably, disclosure of the interviewer's lived experience may have also influenced the level of trust and, subsequently, the degree of disclosure of experiences.

### 4.1 Conclusion

Together, the findings captured interconnected features of the crisis care experience. The interconnection of themes is unsurprising, given that experiences of crisis and crisis care have been understood to be multifaceted and intertwined.^51^ It was clear that EDs were not viewed as appropriate in providing emotional support to people experiencing mental health crises and suicidality. If crisis care providers working in this area are to concern themselves with the best outcomes, particularly in ED, their focus cannot solely be on medical needs. Emotional containment was especially needed when life seemed too much to bear. While short‐term physical containment can help resolve life‐threatening medical needs, it cannot satisfy psychological needs that underpin the mental health crisis, which may threaten the relevance of any medical care provided. The value of crisis phones lies in their accessibility, and the perceived potential of phone lines could be enhanced through nonstandardised and less risk‐averse crisis support. The findings support the more significant development of crisis alternatives that capitalise on offering welcoming spaces that reduce the need for clinical interventions. Overall, findings can help reshape crisis care and emphasise the importance of emotional safety created through human connection, whether in the setting of hospitals, phone lines or the least ubiquitous but highly valued alternative and peer crisis supports.

## AUTHOR CONTRIBUTIONS

**Helena Roennfeldt**: Conceptualisation; methodology; writing—original draft; investigation; writing—review and editing; formal analysis. **Nicole Hill**: Supervision; writing—review and editing; formal analysis; validation. **Louise Byrne**: Supervision; writing—review and editing. **Bridget Hamilton**: Supervision; writing—review and editing; formal analysis; validation.

## CONFLICT OF INTEREST STATEMENT

The authors declare no conflict of interest.

## Data Availability

The data that support the findings of this study are available from the corresponding author upon reasonable request.
